# Development of Cheaper Embryo Vitrification Device Using the Minimum Volume Method

**DOI:** 10.1371/journal.pone.0148661

**Published:** 2016-02-05

**Authors:** Francisco Marco-Jiménez, Estrella Jiménez-Trigos, Victoria Almela-Miralles, José Salvador Vicente

**Affiliations:** 1 Instituto de Ciencia y Tecnología Animal, Universidad Politècnica de València, Valencia, Spain; 2 Institute of Biomedical Sciences, Department of Animal Production, Animal Health and Science and Food Technology, Faculty of Veterinary Medicine, CEU-Cardenal Herrera University, Alfara del Patriarca, Valencia, Spain; IGBMC/ICS, FRANCE

## Abstract

This study was designed to compare the efficiency of the Cryotop and Calibrated plastic inoculation loop (CPIL) devices for vitrification of rabbit embryos on in vitro development and implantation rate, offspring rate at birth and embryonic and fetal losses. CPIL is a simple tool used mainly by microbiologists to retrieve an inoculum from a culture of microorganisms. In experiment 1, embryos were vitrified using a Cryotop device and a CPIL device. There were no significant differences in hatched/hatching blastocyst stage rates after 48 h of culture among the vitrified groups (62±4.7% and 62±4.9%, respectively); however, the rates were significantly lower (P<0.05) than those of the fresh group (95±3.4%). In experiment 2, vitrified embryos were transferred using laparoscopic technique. The number of implanted embryos was estimated by laparoscopy as number of implantation sites at day 14 of gestation. At birth, total offspring were recorded. Embryonic and fetal losses were calculated as the difference between implanted embryos and embryos transferred and total born at birth and implanted embryos, respectively. The rate of implantation and development to term was similar between both vitrification devices (56±7.2% and 50±6.8% for implantation rate and 40±7.1% and 35±6.5% for offspring rate at birth); but significantly lower than in the fresh group (78±6.6% for implantation rate and 70±7.2% for offspring rate at birth, P<0.05). Likewise, embryonic losses were similar between both vitrification devices (44±7.2% and 50±6.8%), but significantly higher than in the fresh group (23±6.6%, P < 0.05). However, fetal losses were similar between groups (10±4.4%, 15±4.8% and 8±4.2%, for vitrified, Cryotop or CPIL and fresh, respectively). These results indicate that the CPIL device is as effective as the Cryotop device for vitrification of rabbit embryos, but at a cost of €0.05 per device.

## Introduction

Vitrification was introduced in 1985 as a simple and cheap way to cryopreserve mammalian embryos in the absence of ice [1]. The first successful vitrification was done with mouse embryos using a relatively large volume sample (0.25-mL straw). Shortly after, Arav [2–6] introduced the idea of using the same technique for vitrification in a small drop, which he later designated the “minimum drop size”. However, vitrification of embryos, on the other hand, although initially attempted in the late 1980s, was not applied clinically until recently [7]. The simplicity of the procedures, high viability of recovered embryos and cost effectiveness of the setup has resulted in increasing use of embryo vitrification compared to the conventional slow-freezing cryopreservation method in most embryo conservation programs [8]. Now, vitrification is being widely utilized in livestock and human embryos [8].

Vitrification is currently producing very satisfactory outcomes by means of methodologies that use a minimal volume [9,10]. Today, several vitrification devices are commercially available; electron microscope grid, minimum drop size technique, Cryotop, Cryoloop, Cryolock, Hemi-straw, solid surface, nylon mesh, Cryoleaf, direct cover vitrification, fiber plug, vitrification spatula, Cryo-E, plastic blade, Vitri-Inga, plastic straw, open-pulled straw, closed pulled straw, flexipet-denuding pipette, superfine open-pulled straw, CryoTip, pipette tip, high-security vitrification device, sealed pulled straw, Cryopette, Rapid-i, and JY Straw [7, 11, 12]. Although some devices can be made in-house, the devices designed to reduce the volume are difficult to produce in-house and do not guarantee safe routine application. Perhaps one of the main limitations of these devices is the high cost (each Cryotop costs €20 per device).

Thus, we propose the use of disposable, sterile calibrated plastic inoculation loop (CPIL), a simple tool used mainly by microbiologists to retrieve an inoculum from a culture of microorganisms. The advantages of this device are that it is calibrated, commercially available, in individual and sterile packages and at a significantly lower cost (approximately €0.05 per device).

This study was therefore designed to compare the efficacy of the CPIL and Cryotop devices for embryo vitrification by analyzing the subsequent in vitro development and live offspring rate at birth in rabbit.

## Materials and Methods

All chemicals, unless otherwise stated, were reagent- grade and purchased from Sigma-Aldrich Química S.A. (Alcobendas, Madrid, Spain). All the experimental procedures used in this study were performed in accordance with Directive 2010/63/EU EEC for animal experiments and were reviewed and approved by the Ethical Committee for Experimentation with Animals of the Polytechnic University of Valencia, Spain (research code: 2015/VSC/PEA/00061).

### Animals

New Zealand White rabbits were used. The rabbit has been used as an experimental animal in genetics and reproduction physiology since the beginning of the century [13]. The great advantage of rabbit is that it is one of the few species in which ovulation is induced by mating, resulting in an exactly defined pregnancy and embryonic age (hours or days post coitum) [13].

Animals were housed at the Polytechnic University of Valencia experimental farm in flat deck indoor cages (75×50×30 cm), with free access to water and commercial pelleted diets (minimum of 15 g of crude protein per kg of dry matter (DM), 15 g of crude fiber per kg of DM, and 10.2 MJ of digestible energy (DE) per kg of DM). The photoperiod is set to provide 16 h of light and 8 h of dark, and the room temperature is regulated to keep temperatures between 10°C and 28°C.

### Embryo collection

A total of 40 nulliparous female animals were used. Female animals were treated with 25 IU of eCG intramuscular (Intervet International B.V., Bowmeer, Holland) to induce receptivity. After 48 hours, female animals were artificially inseminated with a heterospermic pool of semen from male animals of the same line to randomize male effect. At the time of artificial insemination, female animals were administered 1 μg of buserelin acetate (Hoechst Marion Roussel S.A., Madrid, Spain) to induce ovulation and euthanized 72 hours later. Embryos were collected at room temperature by flushing the oviducts and uterine horns with 10 mL of embryo recovery media consisting of Dulbecco phosphate buffered saline (DPBS) supplemented with 0.2% (wt/vol) bovine serum albumin (BSA) and antibiotics (penicillin G sodium 60IU/mL, penicillin G procaine 140IU/mL and dihydrostreptomycin sulfate 0.250 mg/mL; Penivet 1; Divasa Farmavic, Barcelona, Spain). After recovery, morphologically normal embryos (morulae and blastocysts, S1 Fig) were classified as normal (presenting homogenous cellular mass, mucin coat and spherical zona pellucida according to International Embryo Transfer Society classification) and pooled to randomize embryo effect.

### Vitrification and warming procedure

**Fig 1:**
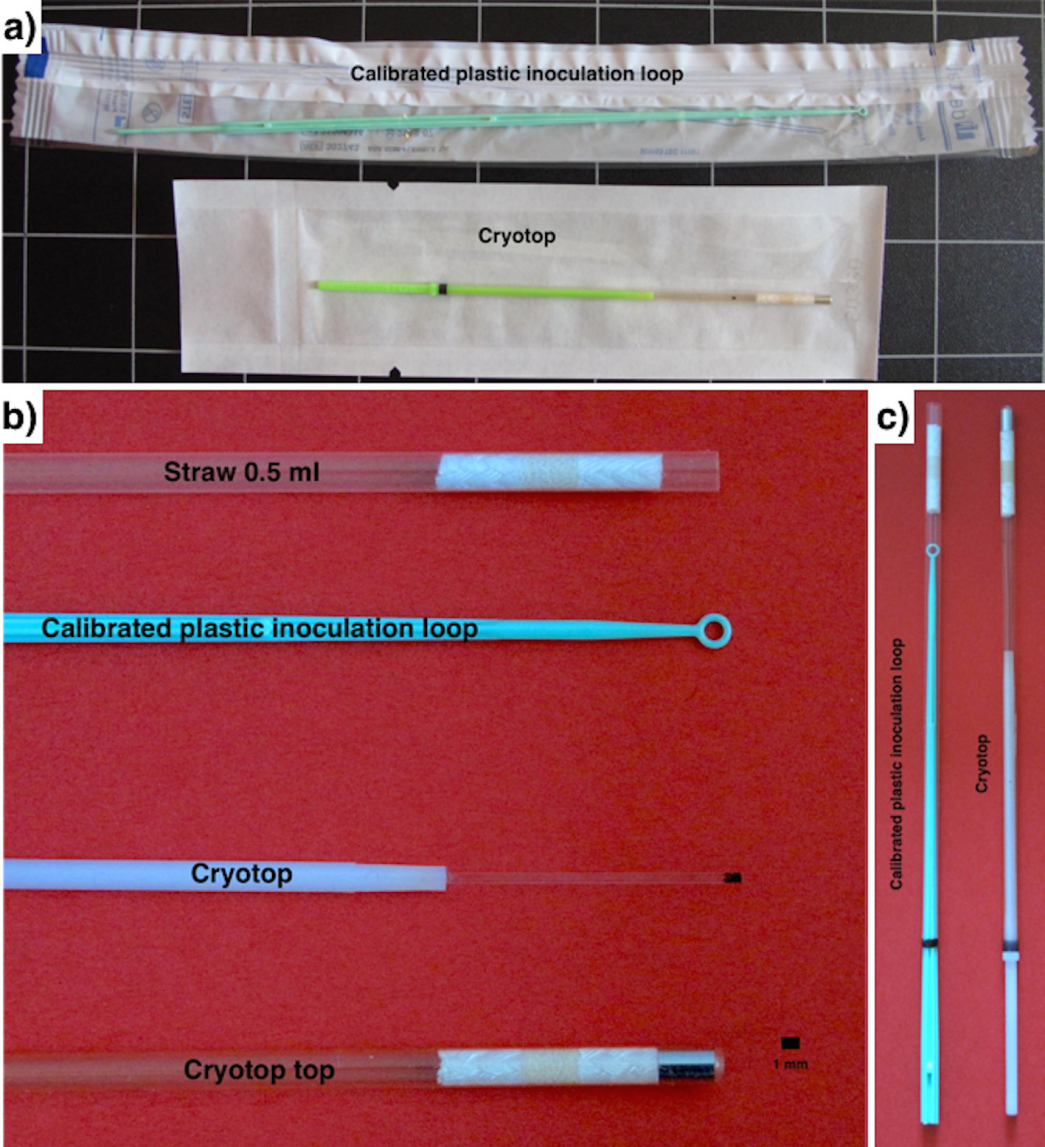
Image shows calibrated plastic inoculation loop and Cryotop devices. a) Both devices packaged in individual sterilized bag. b) Shows each device with the corresponding covers. c) Detail of the device covers.

Embryos were vitrified using the vitrification procedure described by Marco-Jiménez et al. [14] using two devices (Fig 1): Cryotop (Kitazato Co., Fuji, Japan) and Calibrated plastic inoculation loop (CPIL, DELTALAB, Rubí, Spain). The Cryotop consists of a flat rectangular leaf of polypropylene measuring 20 x 0.7 x 0.1 mm attached to a thin, 5-cm long handle [9,15]. Moreover, the thin strip is covered with a hard plastic cover (3 cm long) on top of the Cryotop sheet to protect it during storage in nitrogen containers. The inoculating loop, a disposable sterile plastic loops manufactured from soft-flexible plastic, is a simple tool used mainly by microbiologists to retrieve an inoculum from a microorganism culture. Specifically, we used a calibrated 1μL plastic disposable inoculating loop.

**Fig 2:**
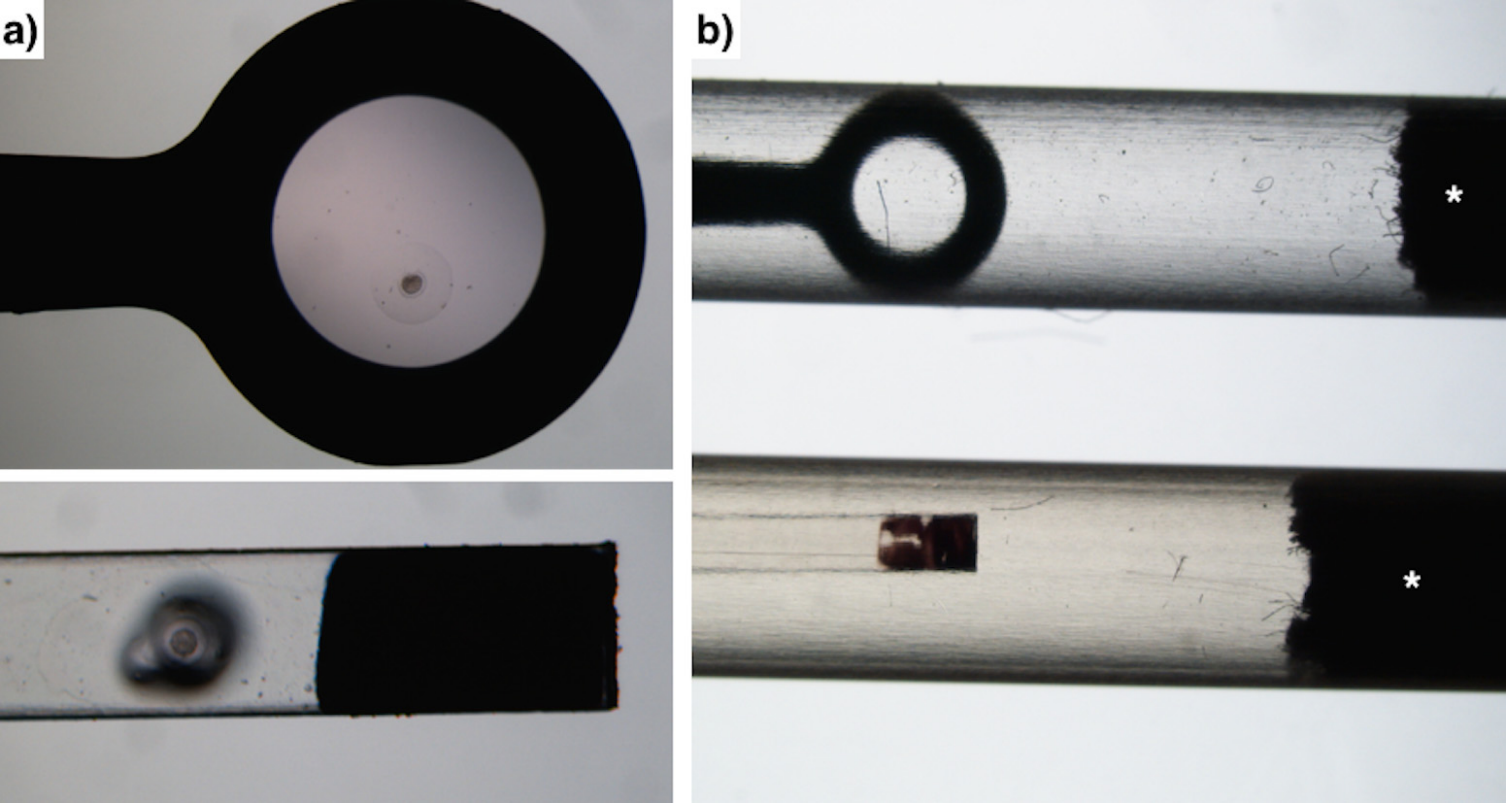
Details of Calibrated plastic inoculation loop and Cryotop devices. a) Magnified image shows embryos in the corresponding devices. b) Shows each device with the corresponding covers (asterisks cotton top).

Embryos were vitrified in a two-step addition procedure. At vitrification time, embryos were transferred into equilibration solution consisting of 10% (vol/vol) ethylene glycol and 10% (vol/vol) dimethyl sulfoxide dissolved in base medium (BM; DPBS supplemented with 0.2% [wt/vol] BSA) at room temperature for 2 minutes. The embryos were then transferred to vitrification solution consisting of 20% (vol/vol) ethylene glycol and 20% (vol/vol) dimethyl sulfoxide in BM. Next, the embryos were loaded into the devices (Fig 2) and directly plunged into liquid nitrogen within 1 minute.

After storage in liquid nitrogen, embryos were warmed by abrupt immersion of the naked devices in 200-μL drops of 0.33 M sucrose at 25°C in BM; after 5 minutes, the embryos were washed in BM. Warming embryos were scored and only undamaged embryos were catalogued as culturable-transferable.

### Effects of vitrification device on *in vitro* development

A total of 205 vitrified embryos (107 from CPIL and 98 from Cryotop) and 41 non-vitrified (fresh embryos) were cultured for 48 h in medium TCM199 containing 10% (v⁄v) Fetal Bovine Serum (FBS) at 38.5°C and 5% CO2 in humidified atmosphere. The in vitro development ability until hatching/hatched blastocyst stage was recorded for analysis.

### Effects of vitrification device on implantation rate, offspring rate at birth and embryonic and fetal losses

A total of 102 vitrified embryos (48 from CPIL and 54 for Cryotop and) and 40 fresh embryos were transferred into 12 adult nulliparous females. Only receptive females (determined by vulva color) were induced to ovulate by injection of 1 μg of buserelin acetate (Hoescht, Marion Roussel, Madrid, Spain), 64–66 hours before transfer. To sedate the does during laparoscopy, anaesthesia was administered by an intramuscular injection of 4 mg/Kg of xylazine (Bayer AG, Leverkusen, Germany), followed 5–10 min later following intravenous injection into the marginal ear vein of 0.4 ml/ Kg of weight of ketamine hydrochloride (Imalgène 500, Merial SA, Lyon, France). During laparoscopy, 3 mg/kg of morphine hydrochloride (Morfina, B. Braun, Barcelona, Spain) was administered intramuscularly. After transfer, does were treated with antibiotics (4mg/Kg of gentamicin every 24h for 3 days, 10% Ganadexil, Invesa, Barcelona, Spain) and analgesics (0.03mg/Kg of buprenorphine hydrochloride, [Buprex, Esteve, Barcelona, Spain] every 12 hours for 3 days and 0.2mg/Kg of meloxicam [Metacam 5mg/mL, Norvet, Barcelona, Spain] every 24h for 3 days). Embryo transfer was performed using the laparoscopic technique described by Besenfelder and Brem [16] (S2 Fig). The number of embryos transferred was 10–12 (six embryos into each oviduct).

Survival rates of vitrified embryos were assessed by laparoscopy following the previous procedure, noting implantation rate (number of implanted embryos at day 14 from total embryos transferred) and birth rate (offspring born/total embryos transferred). Embryonic losses were calculated as the difference between embryos transferred and implanted embryos. Fetal losses were calculated as the difference between total born at birth and implanted embryos.

### Statistical analysis

A generalized linear model including the vitrification devices (Cryotop and CPIL) as fixed effects was used. The error was designated as having a binomial distribution using probit link function. Binomial data for implantation rate, offspring rate at birth and fetal losses were assigned as 1 if positive development had been achieved or a 0 if it had not. A P value of less than 0.05 was considered to indicate a statistically significant difference. The data are presented as least square mean ± standard error mean. All statistical analyses were carried out using a commercially available software program (SPSS 21.0 software package; SPSS Inc., Chicago, Illinois, USA, 2002).

## Results

As shown in Table 1, rates of embryos developing to the hatched blastocyst stage after 48h of culture were similar between vitrified groups, but significantly lower than in the fresh group (62±4.7% and 62±4.9% vs 95±3.4%, for vitrified using Cryotop and CPIL device vs fresh, respectively, P<0.05).

**Table 1:** Effect of vitrification device after 48 h of in vitro culture.

Experimental group	n	Hatching/Hatched Blastocyst rate
Cryotop	98	62±4.7^b^
Calibrated plastic inoculation loop	107	62±4.9^b^
Fresh	41	95±3.4^a^

n: number of embryos.

a,b: Data in the same column with uncommon letters are different (p < 0.05). Data are presented as least squares means ± standard error of the least squares means.

The rate of implantation and development to term was significantly lower in the vitrified groups than in the fresh control (56±7.2% and 50±6.8% vs 78±6.6% for implantation rate and 40±7.1% and 35±6.5% vs 70±7.2% for offspring rate at birth, for vitrified, Cryotop or CPIL vs fresh, respectively, P < 0.05, Table 2), with no differences between the groups vitrified. Embryonic losses were significantly higher in the vitrified groups than in the fresh control (44±7.2% and 50±6.8% vs 23±6.6%, for vitrified, Cryotop or CPIL vs fresh, respectively, P < 0.05, Table 2); there were no differences between vitrified groups. However, fetal losses were similar between groups (10±4.4%, 15±4.8% and 8±4.2%, for vitrified, Cryotop or CPIL and fresh, respectively, Table 2).

**Table 2:** Effect of vitrification device on implantation, offspring at birth and embryonic and fetal losses.

Experimental group	n	Implantation rate	Offspring at birth rate	Losses rate	
				Embryonic^#^	Fetal^*^
Cryotop	54	56±7.2^b^	40±7.1^b^	44±7.2^b^	10±4.4
Calibrated plastic inoculation loop	48	50±6.8^b^	35±6.5^b^	50±6.8^b^	15±4.8
Fresh	40	78±6.6^a^	70±7.2^a^	23±6.6^a^	8±4.2

n: number of embryos.

# Calculated as differences between transferred embryos and implanted embryos.

* Calculated as differences between implanted embryos and offspring at birth.

a,b: Data in the same column with uncommon letters are different (p < 0.05). Data are presented as least squares means ± standard error of the least squares means.

## Discussion

Many techniques have been developed to reduce sample volume with an explosion of methods appearing in the literature over the last decade [7]. However, devices designed to reduce the volume of cryoprotectant required for vitrification are difficult to produce in-house and the commercially available kits are rather expensive (cost approximately €20 per device). The presented study described the use of a device for embryo vitrification in minimum volume size, more importantly commercially available, sterilized and at a low cost of €0.05 per device. To the best of our knowledge, we also report the first offspring born using this new device.

CPIL is a simple tool used mainly by microbiologists to retrieve an inoculum from a culture of microorganisms. CPILs are molded from medical grade polypropylene and sterilized by ethylene oxide gas. They are also free of lubricants, oils and electrostatic charges. The CPILs are enclosed, individually wrapped inside medical style paper–plastic peel pouches or peel-apart packages. Moreover, the loop can be covered with a hard plastic cover (3 cm long using a 0.5 ml sperm straws, Fig 2) on top of the CPIL sheet to protect it during storage in nitrogen containers in accordance with the regulatory directive of the European Union [17]. Recently, Mikołajewska et al. [18] compared the survival rate of matured cat oocytes vitrified with the use of Cryotop and plastic Cryoloop and found no statistical difference.

Over the last decade, researchers have developed a series of devices that permit cooling at 10 to 20 times the rates achievable with 0.25 ml insemination straws immersed in LN_2_ [19]. The devices achieve these results primarily because they have a low thermal mass and because they use very small volumes of cell suspensions. Cryotop with 0.1 μl of volume cools at a mean of 69,250 ± 4,280°C/min from 20°C to −120°C when immersed directly into LN2 and warms from −170°C to −30°C at a mean of 117,500 ± 10,630°C/min when abruptly transferred from LN2 into a 2 ml 0.5 M sucrose solution “bath” at 23°C [19]. Based on the results, the Cryotop method is considered the gold standard for vitrification [20]. Cryotop results in high survival and developmental rates in the vast majority of species; pigs [21–23], cattle [9, 24,25], buffalo [25–27], mice [28], rabbits [14,29] and humans [30–34]. Thus, the Cryotop device was chosen as the comparison point with CPIL device due to its superiority compared to the rest of the devices.

In the present study, vitrified embryos using both devices, Cryotop and CPIL, had similar *in vitro* hatching rates, which were similar to those previously reported [35, 36]. To the best of our knowledge, no studies have addressed this issue using embryos. The only available study used cat oocytes, and no differences were detected in survival rate with plastic inoculation loop compared to Cryotop [18]. When performing the embryo transfer experiment, we confirmed that CPIL and Cryotop were equally effective in terms of pregnancy rate and offspring at birth. Similar results have been reported previously [14, 35–44]. Therefore, these results prove the effectiveness of the calibrated plastic inoculation loop as vitrification device. In addition, the results of our comparison study of the effect of cryodevices on embryonic and fetal losses showed that there was no significant difference between the CPIL and Cryotop. However, the embryonic losses were higher in the vitrified groups than the fresh group. Our data showed that after vitrification one peak of loss occurs before implantation, but after implantation till the end of gestation, both devices and fresh group followed similar paths. The results of the distribution of losses are in accordance with previous studies [14, 42–44].

We conclude that the calibrated plastic inoculation loop device can be applicable with rabbit embryos and suggest that it could also be widely applicable in others species and in a variety of areas including animal industries, experimental animal breeding, conservation biology and reproductive medicine, at negligible cost (0.05€ per device). Further studies should be performed to evaluate the production of live offspring from oocytes vitrified by the calibrated plastic inoculation loop device.

## Supporting Information

S1 Fig Morphologically normal embryos (morulae and blastocysts) catalogued as culturable-transferable. (TIFF)

S2 Fig Representative embryo oviductal transfer assisted by laparoscopy. Epidural catheter introduced into the inguinal region with an epidural needle (A). Embryos were aspirated in an epidural catheter and it was inserted in the oviduct through the infundibulum (B, C & D). Detail of fluid after transfer (E & F).
(TIFF)
