# Supplementary figures and images for: Development of Cheaper Embryo Vitrification Device Using the Minimum Volume Method
Source: PLoS One. 2016 Feb 5;11(2):e0148661. doi: 10.1371/journal.pone.0148661 (PMC4743988; doi:10.1371/journal.pone.0148661)

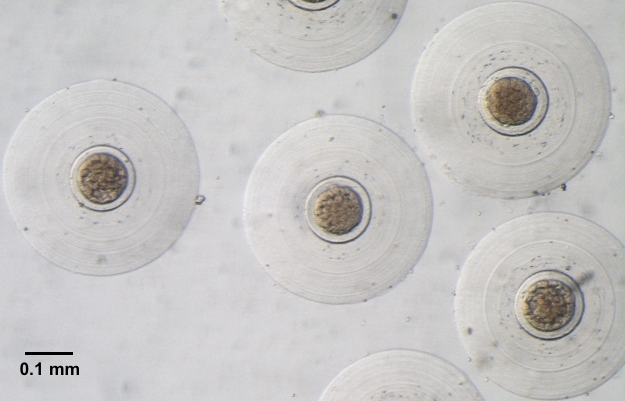

Supplement: S1 Fig — (TIFF) [file pone.0148661.s001.tiff]

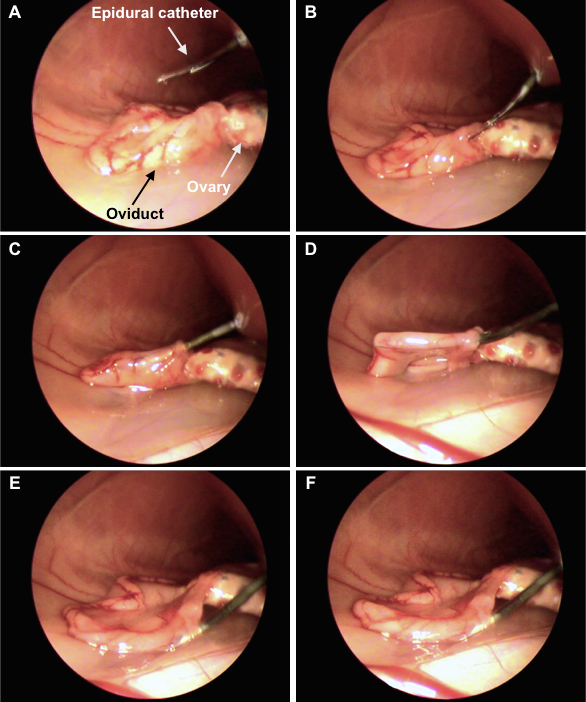

Supplement: S2 Fig — Epidural catheter introduced into the inguinal region with an epidural needle (A). Embryos were aspirated in an epidural catheter and it was inserted in the oviduct through the infundibulum (B, C & D). Detail of fluid after transfer (E & F). (TIFF) [file pone.0148661.s002.tiff]
